# Herp regulates intracellular survival of *Mycobacterium tuberculosis* H37Ra in macrophages by regulating reactive oxygen species-mediated autophagy

**DOI:** 10.1128/mbio.01535-23

**Published:** 2023-10-06

**Authors:** Sang-Hun Son, Junghwan Lee, Soo-Na Cho, Ji-Ae Choi, Jaewhan Kim, Tam Doan Nguyen, Seong-Ahn Lee, Doyi Son, Chang-Hwa Song

**Affiliations:** 1 Department of Microbiology, College of Medicine, Chungnam National University, Daejeon, South Korea; 2 Department of Medical Science, College of Medicine, Chungnam National University, Daejeon, South Korea; 3 Translational Immunology Institute, Chungnam National University, Daejeon, South Korea; Leibniz Institute for Natural Product Research and Infection Biology-Hans Knoell Institute Jena (HKI), Jena, Germany; Francis Crick Institute, London, United Kingdom

**Keywords:** ER stress, *Mycobacterium tuberculosis*, homocysteine-inducible ER protein with ubiquitin-like domain 1, reactive oxygen species, ER-associated degradation, autophagy

## Abstract

Novel treatment strategies for tuberculosis (TB), such as host-directed therapeutics, may offer therapeutic options for patients with drug-resistant TB. Endoplasmic reticulum (ER) stress-mediated apoptosis is one of the host defense mechanisms used to remove mycobacteria. It is reported that homocysteine-inducible ER protein with ubiquitin-like domain 1 (Herp) inhibits apoptosis by preventing the loss of ER Ca^2+^ and mitochondrial potential during ER stress. However, the roles of Herp in ER stress and apoptosis during H37Ra infection are largely unknown. Here, we show that Herp is induced in H37Ra-infected macrophages through an activating transcription factor 6 (ATF6)-dependent ER stress response. Suppressing Herp by genetic approaches decreased production of HRD1, conserved branch of mammalian ER-associated degradation (ERAD) machinery, and increased the production levels of ER stress-associated molecules such as p-IRE1ɑ and BiP after H37Ra infection. Suppressing Herp also increased both the NADPH oxidase 2 and inositol triphosphate receptor, which sequentially led to increased reactive oxygen species (ROS) production during H37Ra infection. Interestingly, the Herp depletion-mediated ROS increment led to autophagy induction, which led to decreased intracellular survival of mycobacteria in H37Ra-infected macrophages. The role of Herp was further confirmed by the fact that blocking this molecule *in vitro* and *in vivo* significantly reduced mycobacterial survival. These findings indicate that Herp mediates crosstalk between ER stress and ROS-mediated autophagy during H37Ra infection, suggesting the potential of Herp manipulation as a therapeutic strategy for *Mycobacterium tuberculosis* (Mtb) infection.

**IMPORTANCE:**

Several studies have suggested that endoplasmic reticulum (ER) stress is important in the pathogenesis of infectious diseases; however, the precise function of ER stress regulation and the role of Herp as a regulator in Mtb H37Ra-induced ER stress remain elusive. Therefore, our study investigated ER stress and autophagy associated with Herp expression in *Mycobacterium tuberculosis*-infected macrophages to determine the role of Herp in the pathogenesis of tuberculosis.

## INTRODUCTION

*Mycobacterium tuberculosis* (Mtb) is a human pathogen responsible for several million deaths annually (1). Therefore, there is an urgent need to develop new therapeutic drugs to control tuberculosis (TB) (2). Host-directed therapy (HDT) may enhance the effectiveness of TB treatment (3, 4) by boosting the host’s immune system rather than directly targeting mycobacteria, making it a promising approach for improving treatment outcomes.

There are two widely used laboratory reference strains: Mtb virulent strains H37Rv and avirulent strains H37Ra. It is well known that Mtb regulates cell death to survive in host cells (5). H37Ra infection relatively increases endoplasmic reticulum (ER) stress-related molecules and triggers apoptosis in macrophages compared to H37Rv (6 – 8). On the other hand, H37Rv infection leads to necrosis and decreases ER stress-related molecules compared to H37Ra in macrophages (7 – 9). Despite these differences, both H37Rv and H37Ra induce apoptotic cell death and ER stress in macrophages (8, 10, 11). In this study, we used H37Ra to reveal the function of ER stress-mediated apoptosis for suppression of intracellular mycobacteria because it induced ER stress-mediated apoptosis more strongly than H37Rv.

The ER is generally responsible for protein synthesis, folding, modification, and transport (12). ER stress is involved in various host cell responses during bacterial infections (13). A previous study suggested that ER stress alleviates lung injury by the activation of GSK-3β and XBP-1 in infections caused by *Pseudomonas aeruginosa* (14). *Brucella melitensis* infections induce unfolded protein response (UPR) to promote intracellular bacterial growth (15), while *Simkania negevensis* infections inhibit UPR to promote its intracellular proliferation (16). Our previous studies also suggest that Mtb-induced ER stress plays an important role in the development of TB (10, 17). Although several studies have suggested that ER stress is important in the pathogenesis of infectious diseases, the precise function of ER stress regulation must be investigated at the molecular level.

Homocysteine-inducible ER protein with ubiquitin-like domain 1 (Herp), which is localized in the ER membrane, has been reported to be involved in the protection of cells against ER stress (18). Herp inhibits apoptosis by preventing the loss of ER Ca^2+^ and mitochondrial potential during ER stress (19). The binding of Herp regulates hydroxymethyl glutaryl-coenzyme A reductase degradation protein 1 (Hrd1)-mediated ubiquitylation in ER-associated protein degradation (ERAD), which results in cell survival during ER stress (20 – 22). Previous reports suggested that Herp inhibits apoptosis by preventing the loss of ER Ca^2+^ and mitochondrial potential during ER stress, thereby preventing the progression of Parkinson’s disease (19, 23, 24). In this study, we investigated ER stress and autophagy associated with Herp expression in Mtb-infected macrophages to determine the role of Herp in the pathogenesis of TB, in which ER stress is implicated.

Autophagy is a conserved catabolic process that recycles intracellular components to provide nutrients during starvation and for protein quality control (25). In addition to cellular homeostasis, autophagy is required for the elimination of pathogens, such as viruses, parasites, and bacteria (26). Autophagy in macrophages accelerates the maturation of mycobacteria-containing phagosomes and increases lysosome-mediated mycobacterial killing (27). Autophagic elimination of intracellular bacteria is largely dependent on targeting bacteria with ubiquitin chains (28). Ubiquitin-binding autophagy adaptors are recruited to ubiquitin-associated bacteria and bind to the autophagosomal membrane-associated protein LC3, resulting in the delivery of intracellular bacteria to lysosomes for degradation (28). ER stress has also been shown to induce autophagy (29, 30). Further investigation is necessary to clarify whether the regulatory mechanism of ER stress is involved in the activation of autophagy in response to ER stress.

Although Herp is involved in various cellular processes and has multiple functions, including protein ubiquitylation (22, 31) and ERAD (22), its role in Mtb-induced ER stress remains elusive. In this study, we focused on the role of Herp as a regulator of ER stress during Mtb infections because the regulation of ER stress might be a valuable strategy in HDT against MDR-TB.

## RESULTS

### H37Ra induces Herp production in an activating transcription factor 6 (ATF6)-dependent manner

**Fig 1:**
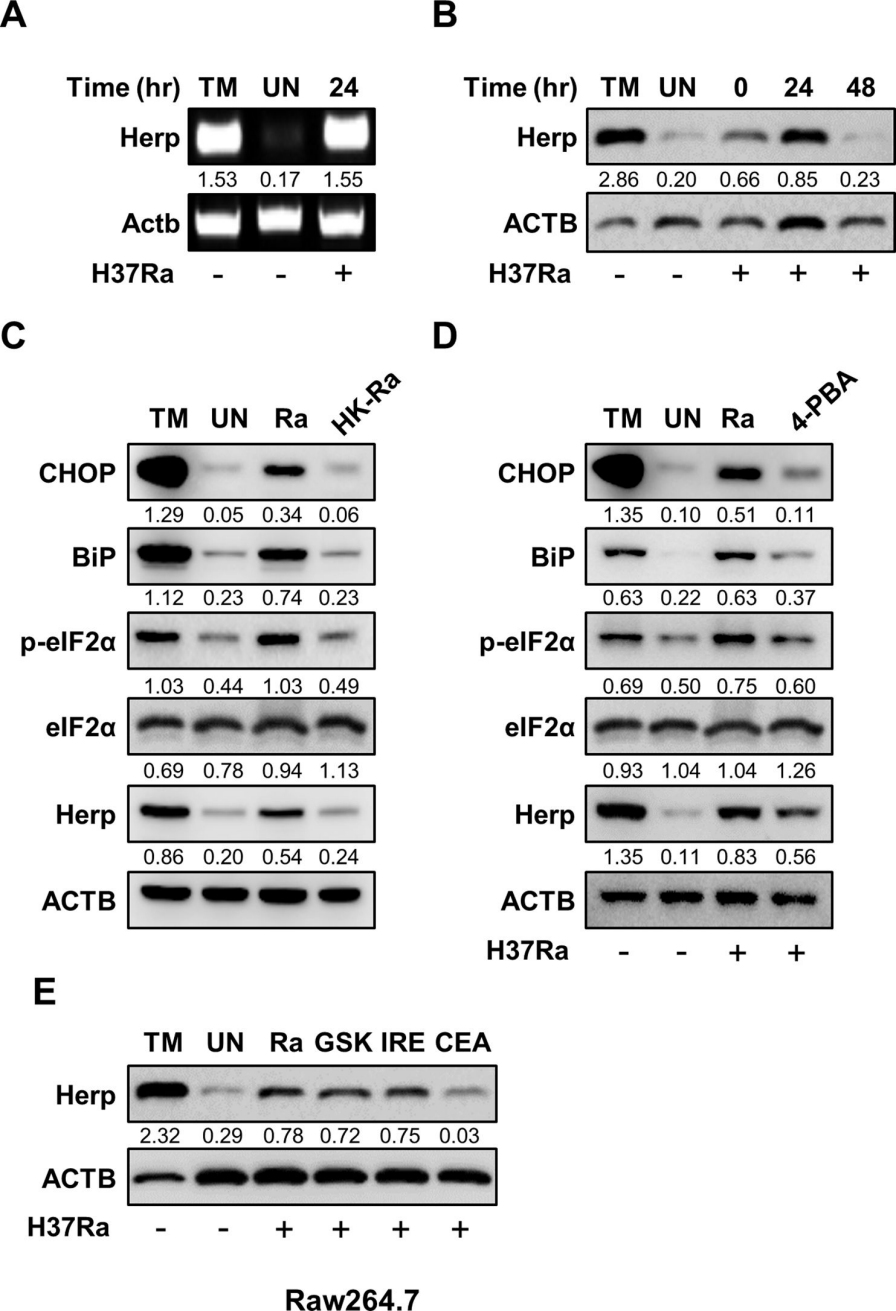
Mtb H37Ra induces Herp production in an ATF6-dependent manner. (**A**) BMDMs were treated with tunicamycin (1 µM), a potential endoplasmic reticulum (ER) stress inducer, for 6 h or infected with H37Ra [multiplicity of infection (MOI ) = 5] for 24 h. Herp mRNA levels were analyzed using polymerase chain reaction. (**B**) BMDMs were infected with H37Ra (MOI = 5) at the indicated times. The untreated control was cultured for 48 h. Protein levels were analyzed using western blotting. (**C**) Raw264.7 cells were infected with H37Ra (MOI = 5) or heat-killed H37Ra (MOI = 5) for 24 h. Protein levels were analyzed using western blotting. (**D**) Raw264.7 cells were pretreated with 4-phenylbutyrate (10 mM), an ER stress inhibitor, for 1 h and then infected with H37Ra (MOI = 5) for 24 h. Protein levels were analyzed using western blotting. (**E**) Raw264.7 cells were treated with ER stress-specific inhibitors (10 µM GSK2606414, 5 µM IREstatin, and 10 µM Ceapin-A7) for 24 h and infected with H37Ra (MOI = 5) for 24 h. Protein levels were analyzed using western blotting. The quantification of western blots was performed using ImageJ and normalized by the ACTB density. CEA, Ceapin-A7; GSK, GSK2606414; HK-Ra, heat-killed H37Ra; IRE, IREstatin; 4-PBA, 4-phenylbutyrate; TM, Tunicamycin; UN, untreated control.

To investigate whether H37Ra infection induces Herp production in macrophages, we measured the mRNA and protein levels of Herp in macrophages after H37Ra infection. H37Ra infection caused an increase in both the mRNA and protein levels of Herp in bone marrow-derived macrophages (BMDMs) (Fig. 1A and B). Similarly, H37Rv infection induced Herp production in BMDMs (Fig. S1A). Next, we assessed the protein levels of the ER stress sensor molecules in the H37Ra-infected Raw264.7 cells. Live H37Ra infection induced CCAAT/enhancer-binding protein homologous protein (CHOP), phospho-alpha subunit of eukaryotic initiation factor 2 (eIF2) α, binding immunoglobulin protein (BiP), and Herp; however, heat-killed H37Ra did not induce these molecules (Fig. 1C). We also examined whether the inhibition of ER stress affected Herp expression during H37Ra infection in Raw264.7 cells. H37Ra-induced Herp production was decreased by pretreatment with the chemical chaperone 4-phenylbutrate (Fig. 1D). These results suggest that only live H37Ra induces Herp production via the ER stress pathway.

To identify which ER stress pathway is important for inducing Herp expression in H37Ra-infected macrophages, we measured Herp production in the presence of specific inhibitors, such as GSK2606414 (a PERK inhibitor), IREstatin (an IRE1 inhibitor), and Ceapin-A7 (an ATF6 inhibitor). H37Ra-induced Herp production was effectively reduced by Ceapin-A7 treatment in Raw 264.7 cells (Fig. 1E). These data suggest that H37Ra-induced Herp production is dependent on the ATF6 signaling pathway.

### Herp is important for ER quality control during H37Ra infection

**Fig 2:**
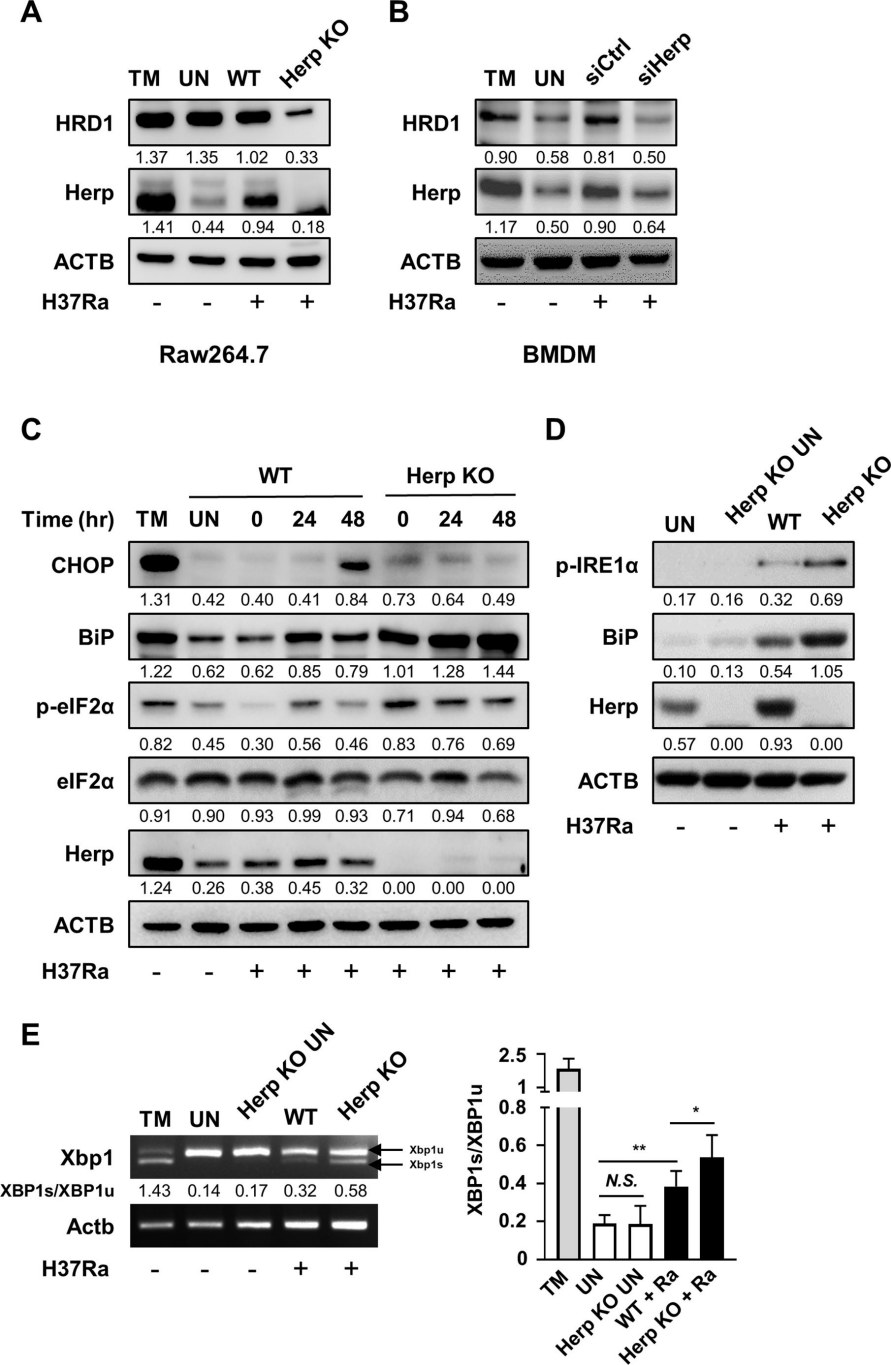
Herp is important for ER quality control during Mtb H37Ra infection. (**A**) Herp-WT and Herp-KO Raw264.7 cells were treated with tunicamycin (1 µM) for 6 h or infected with H37Ra (MOI = 5) for 24 h. Protein levels were analyzed using western blotting. (**B**) BMDMs were transfected with siControl or siHerp and infected with H37Ra (MOI = 5) for 24 h. BMDMs were treated with TM for 6 h. Protein levels were analyzed using western blotting. (**C**) Herp-WT and Herp-KO Raw264.7 cells were infected with H37Ra (MOI = 5) at the indicated times. The untreated control was cultured for 48 h. Protein levels were analyzed using western blotting. (**D**) Herp-WT and Herp-KO Raw264.7 cells were infected with H37Ra (MOI = 5) for 24 h. Protein levels were analyzed using western blotting. (**E**) Herp-WT and Herp-KO Raw264.7 cells were treated with tunicamycin (1 µM) for 6 h or infected with H37Ra (MOI = 5) for 24 h, and mRNA levels were analyzed using PCR. The quantification of western blots was performed using ImageJ and normalized by the ACTB density. Data are expressed as the mean ± standard deviation of three independent experiments (**B**). N.S., not significant (*P* ≥ 0.05). *P < 0.05, **P < 0.01, and ***P < 0.001. TM, Tunicamycin; UN, untreated control.

A previous study suggested that Herp binding to ubiquitin proteins plays an important role in the ERAD pathway (20, 22). Herp has also been known to stabilize the binding of misfolded proteins to the ERAD complex, including HRD1 (32). We investigated whether Herp affects ERAD-associated molecules, such as HRD1, in Herp-depleted macrophages. The protein levels of HRD1 were reduced after H37Ra infection (Fig. 2A and B). The uninfected Herp-knockout (KO) Raw264.7 cells had HRD1 protein levels similar to those of the wild-type (WT) controls (Fig. S2A). Since crosstalk between UPR and ERAD is critical for the maintenance of ER homeostasis (33), we hypothesized that Herp-mediated ER quality control system impairment could increase the ER stress response. We examined whether depleting Herp in macrophages affects the ER stress response to H37Ra infection. Protein levels of p-eIF2α and BiP were increased in H37Ra-infected Herp-KO Raw264.7 cells compared to WT controls (Fig. 2C). Protein levels of p-IRE1α and BiP and mRNA levels of spliced X-box binding protein 1 (XBP1) were also increased in the Herp-KO Raw264.7 cells (Fig. 2D and E). In contrast, CHOP production was lower after 48 h of H37Ra infection in Herp-KO Raw264.7 cells than in WT Raw264.7 cells (Fig. 2C). These differences between WT Raw264.7 cells and Herp-KO Raw264.7 cells were minimal in uninfected Raw264.7 cells (Fig. S2B). All these results indicate that the regulation of Herp affects the H37Ra-mediated ER stress response via ER quality control.

### Herp plays a key role in H37Ra-mediated ROS production and intracellular survival of mycobacteria

**Fig 3:**
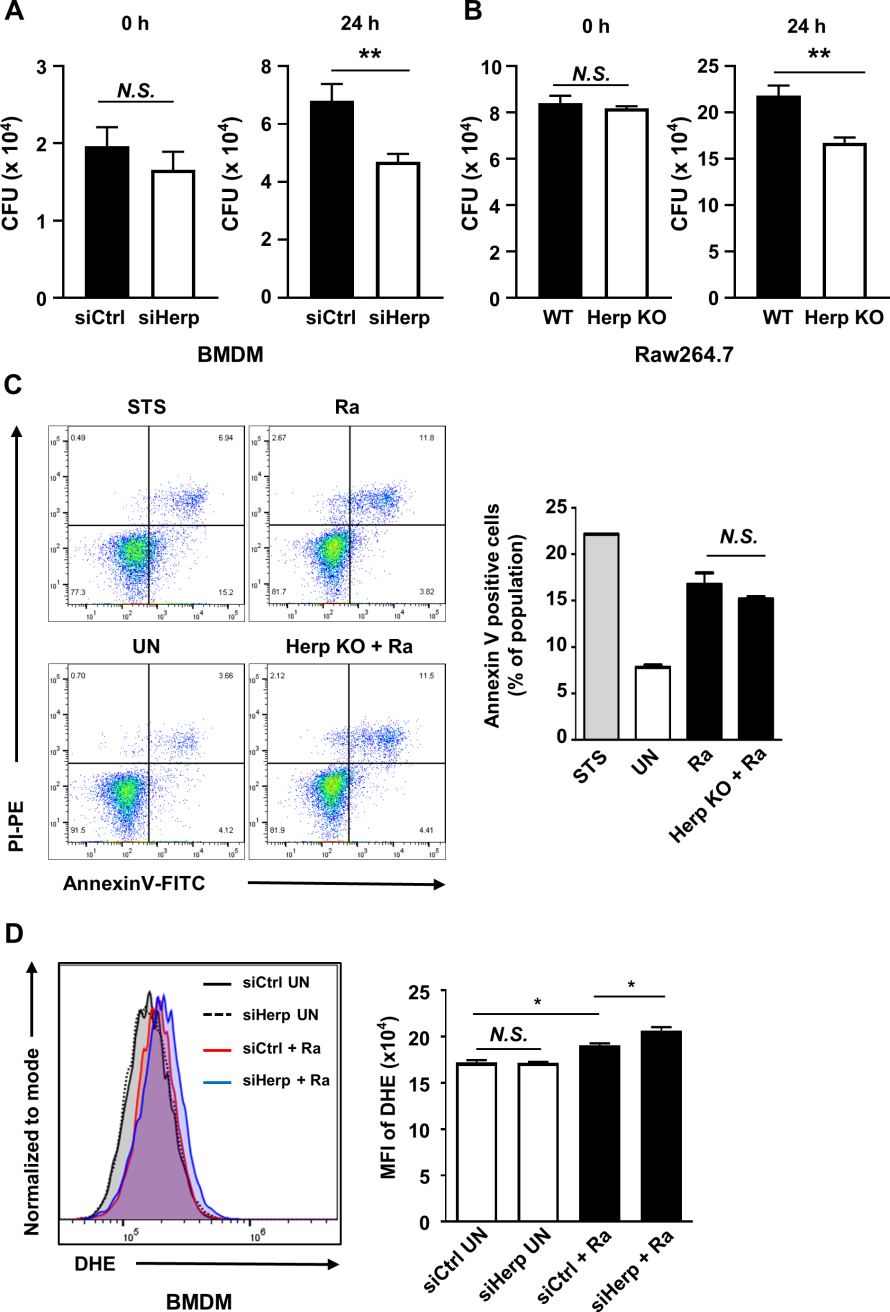
Herp plays a key role in Mtb-mediated ROS production and intracellular survival of mycobacteria. (A) BMDMs were transfected with siControl or siHerp and infected with H37Ra (MOI = 5) for 0 and 24 h. Intracellular survival of H37Ra was measured using a CFU assay. (B) Herp-WT and Herp-KO Raw264.7 cells were infected with H37Ra (MOI = 5) for 0 and 24 h. The intracellular survival of H37Ra was measured using a CFU assay. (C) Herp-WT and Herp-KO Raw264.7 cells were infected with H37Ra (MOI = 5) for 24 h. Raw264.7 cells were treated with staurosporine (500 nM) for 6 h. Apoptosis was analyzed using Annexin-V/PI staining. Bar graphs depict the percentage of Annexin-V-positive cells. (D) BMDMs were transfected with siControl or siHerp and infected with H37Ra (MOI = 5) for 24 h. Levels of ROS were measured using dihydroethidium staining. Data are expressed as the mean ± standard error of the mean of three independent groups (A, B, and D). Data are expressed as the mean ± standard deviation of three independent experiments (C). N.S., not significant (*P* ≥ 0.05). *P < 0.05, **P < 0.01, and ***P < 0.001. STS, staurosporine; UN, untreated control.

Previous studies have shown that apoptosis mediated by the ER stress pathway contributes to the survival of mycobacteria (10, 34, 35). To test whether Herp regulation affects the intracellular survival of mycobacteria in macrophages, we assessed the survival of H37Ra in Herp-depleted macrophages. Significant suppression of H37Ra growth was observed in Herp-knockdown BMDMs (Fig. 3A; Fig. S3A). Similarly, the intracellular survival of H37Ra decreased in Herp-KO Raw264.7 cells compared to the WT controls (Fig. 3B). The rates of phagocytosis, which were determined by the number of colony-forming units (CFUs) at 0 h, showed no difference between the WT and Herp-depleted macrophages (Fig. 3A and B). Furthermore, we analyzed whether Herp regulation affects H37Ra-induced apoptosis in Raw264.7 cells and found no significant effect of Herp-KO on the process (Fig. 3C). Herp-KO Raw264.7 cells also showed similar apoptosis rates to uninfected WT Raw264.7 cells (Fig. S3B).

**Fig 4:**
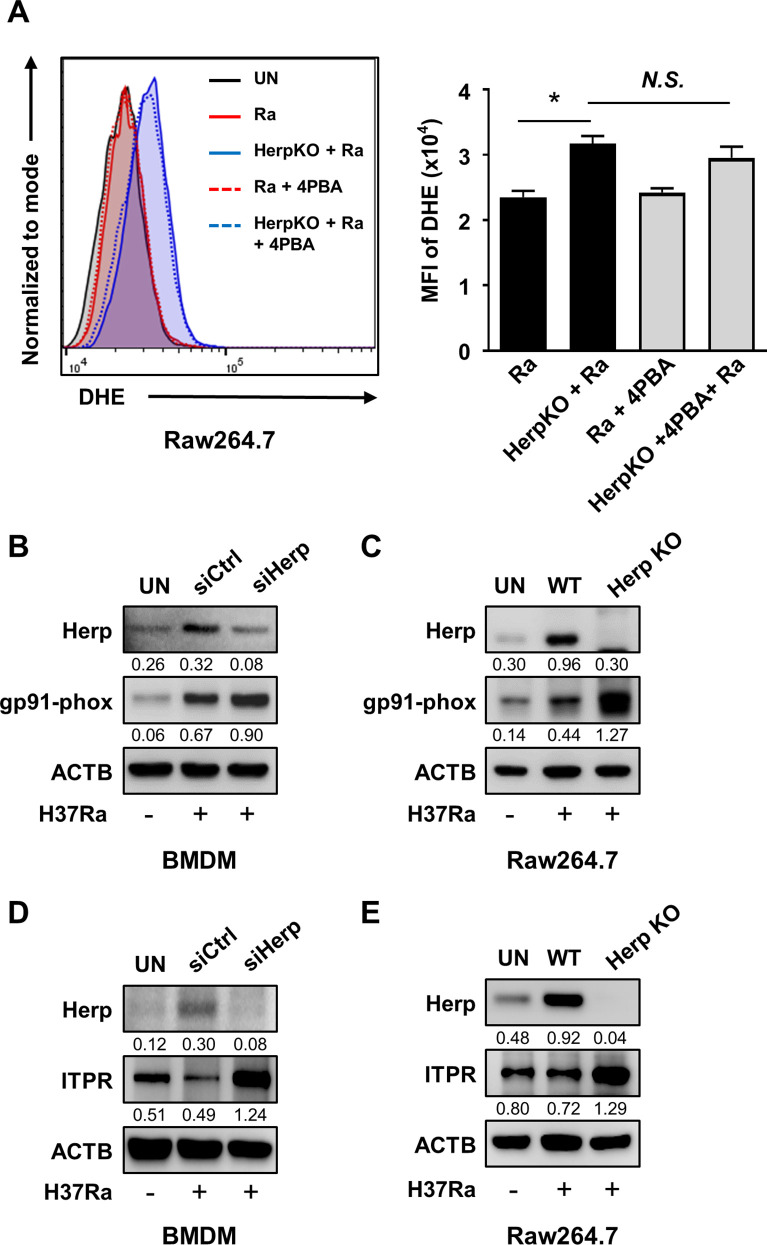
Herp depletion contributes to an increase in gp91-phox and ITPR in Mtb-infected macrophages. (A) Herp-WT and Herp-KO Raw264.7 cells were pretreated with the ER stress inhibitor 4-phenylbutyrate (10 mM) for 1 h and infected with H37Ra (MOI = 5) for 24 h. The levels of ROS were measured by dihydroethidium staining. (B and D) Bone marrow-derived macrophages were transfected with siControl or siHerp and infected with H37Ra (MOI = 5) for 24 h. Protein levels were analyzed using western blotting. (C and E) Herp-WT and Herp-KO Raw264.7 cells were infected with H37Ra (MOI = 5) for 24 h. The protein levels were analyzed using western blotting. The quantification of western blots was performed using ImageJ and normalized by the ACTB density. Data are expressed as the mean ± standard error of the mean of three independent groups (A). N.S., not significant (*P* ≥ 0.05). *P < 0.05, **P < 0.01, and ***P < 0.001. 4PBA, 4-phenylbutyrate; Ra, H37Ra; UN, untreated control.

Next, we examined the relationship between Herp regulation and reactive oxygen species (ROS) synthesis. Herp-knockdown BMDMs showed increased ROS production after H37Ra infection (Fig. 3D). Consistent with this result, ROS production was higher in Herp-KO Raw264.7 cells than in WT Raw264.7 cells during H37Ra infection (Fig. 4A); however, the discrepancy in ROS levels was not significant (Fig. S3C). These results suggest that the regulation of Herp production may affect ROS levels as well as the intracellular survival of bacteria in H37Ra-infected macrophages.

### Herp contributes to the regulation of gp91-phox and inositol triphosphate receptor (ITPR) in Mtb-infected macrophages

To investigate the mechanism by which the loss of Herp increases ROS production in H37Ra-infected macrophages, we determined ROS levels in Herp-KO macrophages infected with H37Ra in the presence or absence of 4-phenylbutyrate compared with that in WT controls. Inhibition of ER stress by 4-phenylbutyrate during mycobacterial infection did not significantly affect ROS levels in the Herp-KO Raw 264.7 cells (Fig. 4A). It has been reported that suppression of the ERAD system leads to the inadequate activity of the ER-localized NADPH oxidase in Yeast (36). Therefore, we investigated whether Herp depletion affected the expression of NADPH oxidase in Herp-depleted macrophages. The protein levels of gp91-phox, an essential component of NADPH oxidase, were increased in Herp-knockdown BMDMs compared to those in the controls (Fig. 4B). Similarly, gp91-phox production increased in H37Ra-infected Herp-KO Raw264.7 cells (Fig. 4C). The uninfected Herp-KO Raw264.7 cells had similar gp91-phox protein levels to the WT controls (Fig. S4A). These data suggest that ROS production may be regulated by the protein levels of NADPH oxidase 2 (NOX2) in Herp-depleted macrophages.

**Fig 5:**
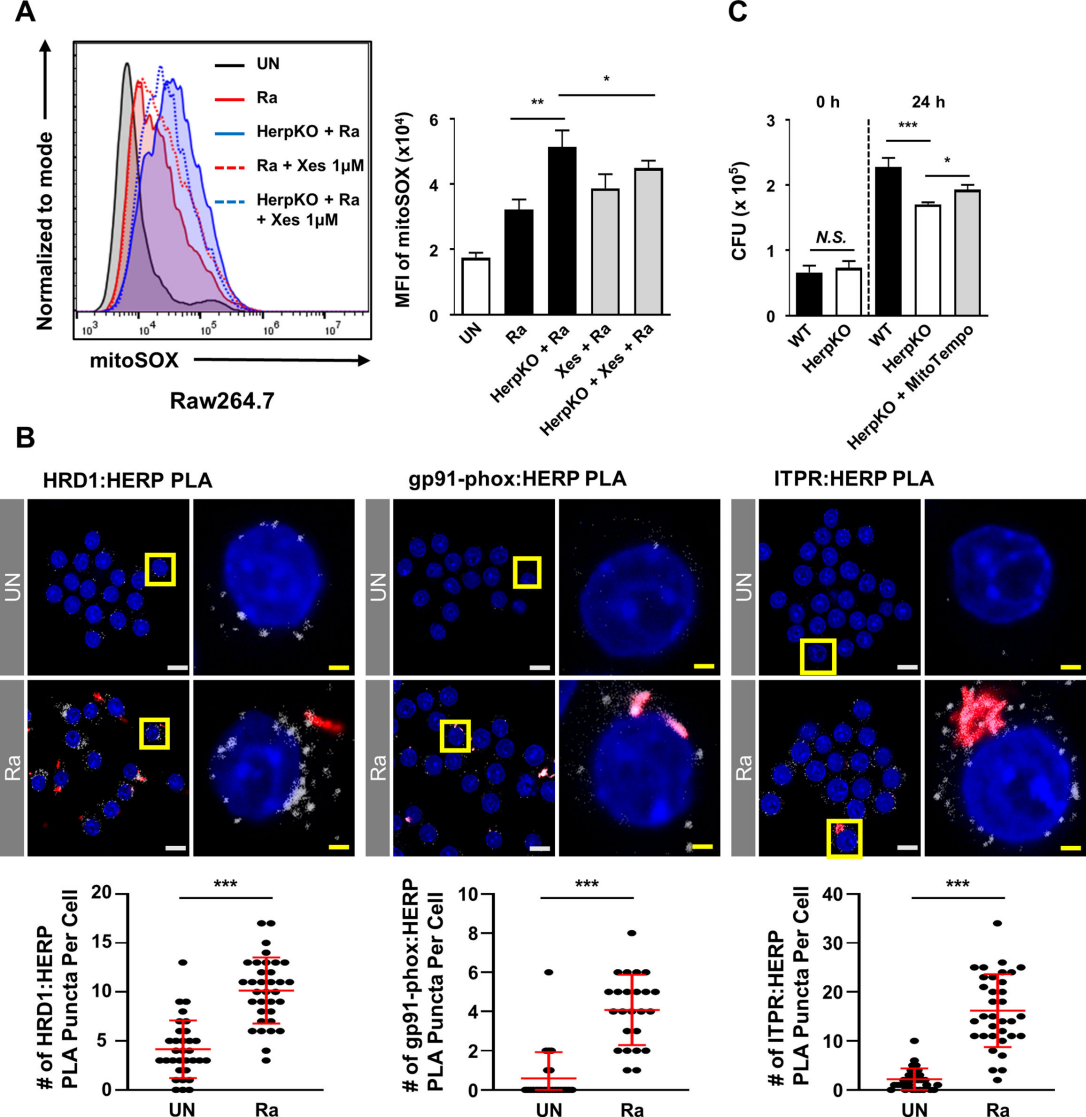
Herp is associated with HRD1, NOX2, and ITPR, while ITPR-mediated mROS suppresses intracellular mycobacteria. (A) Herp-WT and Herp-KO Raw264.7 cells were pretreated with Xestospongin C (1 µM), an ITPR inhibitor, for 1 h and infected with H37Ra (MOI = 5) for 24 h. The levels of mitochondrial ROS were analyzed using MitoSOX staining. (B) Raw264.7 cells were infected with H37Ra (MOI = 5) for 24 h. A PLA was performed between Herp and either HRD1, gp91phox, or ITPR. Representative images of the PLA (white) and quantification of PLA puncta per RFP-positive cell are shown. PLA puncta were counted manually. White scale bars, 10 µM. Yellow scale bars, 2 µM. (C) Herp-WT and Herp-KO Raw264.7 cells were pretreated with MitoTempo (100 µM), a mitochondria-targeted antioxidant, for 3 h and infected with H37Ra (MOI = 5) for 0 and 24 h. The intracellular survival of H37Ra was measured using a CFU assay. Data are expressed as the mean ± standard error of the mean (A and C) or standard deviation (B) of three independent experiments. N.S., not significant (*P* ≥ 0.05). *P < 0.05, **P < 0.01, and ***P < 0.001. Ra, H37Ra; UN, untreated control; Xes, Xestospongin C.

Since overloading of mitochondrial matrix Ca^2+^ leads to increased ROS production (37), and ITPR is involved in cellular Ca^2+^ homeostasis by inducing release of Ca^2+^ from the ER (38), we investigated the protein levels of ITPR, levels of cytosolic Ca^2+^, and mitochondrial ROS (mROS) in Herp-depleted macrophages. The ITPR protein levels were increased in both Herp- knockdown BMDMs and Herp-KO Raw 264.7 cells compared to those in controls (Fig. 4D and E), but they were similar between uninfected Herp-KO Raw264.7 cells and WT controls (Fig. S4A). To investigate the effect of Herp deficiency on the levels of cytosolic Ca^2+^, we measured Fluo-4-AM fluorescence using flow cytometry and fluorescence microscopy. The flow cytometry results showed that the levels of cytosolic Ca^2+^ were increased in H37Ra-infected Herp-KO Raw264.7 cells compared to the WT controls (Fig. S5B). Similar results were obtained with fluorescence microscopy (Fig. S5C). Furthermore, the production of mROS was also increased in H37Ra-infected Herp-KO Raw264.7 cells (Fig. 5A), and the produced mROS was stabilized in Herp-KO Raw264.7 cells upon treatment with Xestospongin C, a selective ITPR antagonist (Fig. 5A). However, the mROS levels in WT Raw264.7 cells were not significantly affected by treatment with Xestospongin C during H37Ra infection (Fig. 5A). Thus, Herp regulation could also affect ITPR-mediated mROS production.

To further validate the regulation of the expression of NOX2 and ITPR proteins by Herp, we investigated putative close associations of proteins, such as HRD1, Herp, NOX2, and ITPR, using a proximity ligation assay (PLA). The binding of Herp to HRD1 regulates the activity of HRD1-containing ERAD complexes (21), indicating that ERAD regulates the proteins that interact with Herp. There was an association between HRD1 and Herp in the Raw264.7 cells (Fig. 5B). PLA puncta were significantly increased in H37Ra-RFP-infected Raw264.7 cells compared to uninfected Raw264.7 cells (Fig. 5B). In addition, our results showed that the interaction of either gp91-phox or ITPR with Herp in Raw264.7 cells increased during H37Ra-RFP infection (Fig. 5B). Notably, we found that MitoTempo, a mitochondria-targeted antioxidant, increased mycobacterial growth in Herp-KO Raw264.7 cells (Fig. 5C). Thus, Herp depletion increased NOX2 and ITPR and affected ROS production in H37Ra-infected macrophages and the intracellular survival of mycobacteria.

### Herp depletion-driven ROS are associated with autophagy during mycobacterial infections

**Fig 6:**
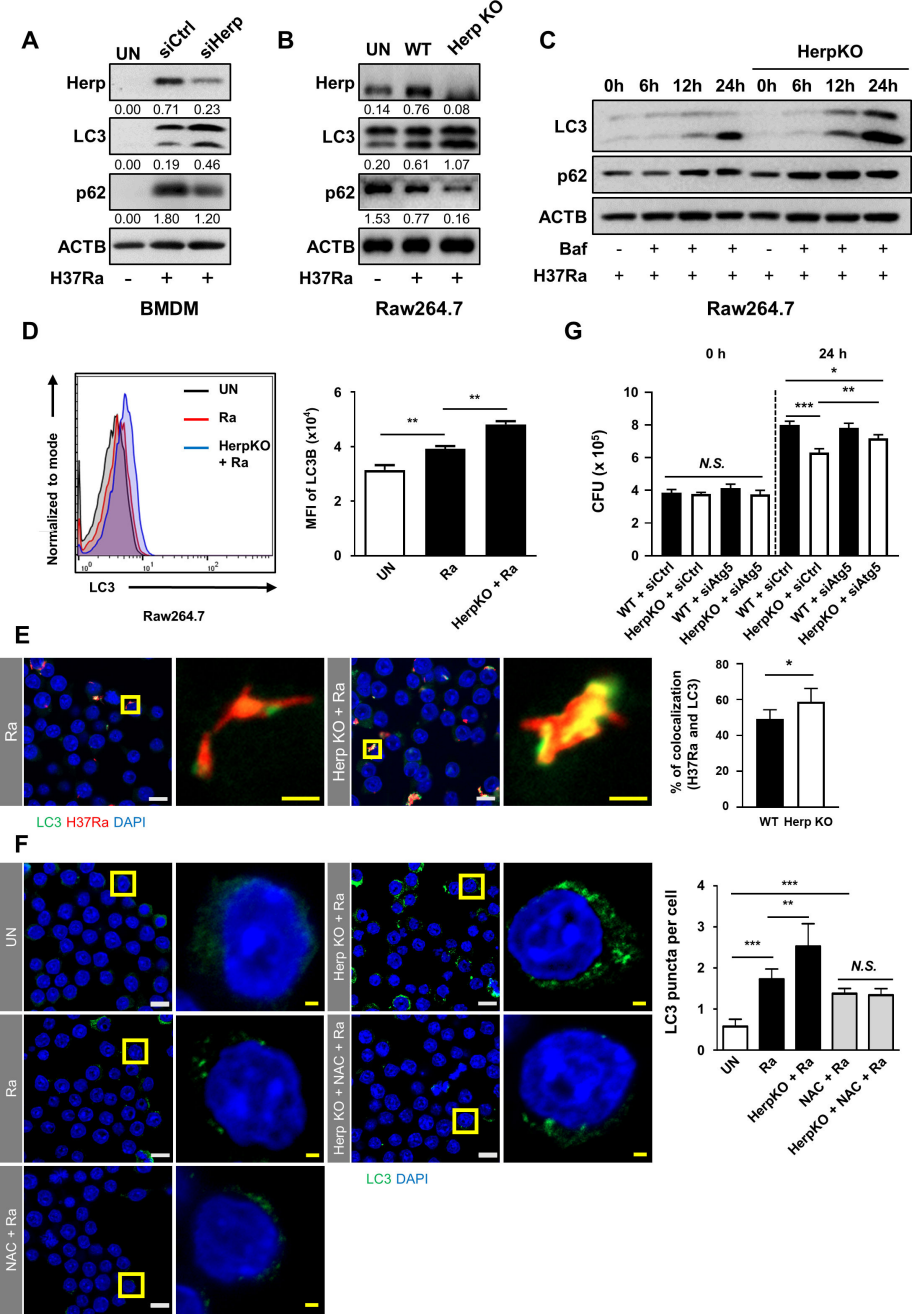
Herp depletion-driven ROS are associated with autophagy during mycobacterial infection. (A) BMDMs were transfected with siControl or siHerp and infected with H37Ra (MOI = 5) for 24 h. Protein levels were analyzed using western blotting. (B) Herp-WT and Herp-KO Raw264.7 cells were infected with H37Ra (MOI = 5) for 24 h. Protein levels were analyzed using western blotting. (C) Herp-WT and Herp-KO Raw264.7 cells were infected with H37Ra (MOI = 5) and then treated with Bafilomycin (1 nM) for the indicated times. Protein levels were analyzed using western blotting. (D) Herp-WT and Herp-KO Raw264.7 cells were infected with H37Ra (MOI = 5) for 24 h. LC3 was analyzed using fluorescence-activated cell sorting. (E) Herp-WT and Herp-KO Raw264.7 cells were infected with H37Ra-RFP (MOI = 5) for 24 h. Cells were stained with anti-LC3 antibody (green) for immunofluorescence. Cell nuclei were visualized using DAPI staining (blue). Quantification of LC3 (green) and H37Ra-RFP colocalization per cell (right). White scale bars, 10 µM. Yellow scale bars, 2 µM. (F) Herp-WT and Herp-KO Raw264.7 cells were infected with H37Ra (MOI = 5) for 24 h. The cells were stained with anti-LC3 antibody (green) for immunofluorescence. Cell nuclei were visualized using DAPI staining (blue). White scale bars, 10 µM. Yellow scale bars, 2 µM. (G) Herp-WT and Herp-KO Raw264.7 cells were transfected with siControl or siAtg5 and infected with H37Ra (MOI = 5) for 0 and 24 h. The intracellular survival of H37Ra was measured using a CFU assay. The quantification of western blots was performed using ImageJ and normalized by the ACTB density. Data are expressed as the mean ± standard deviation of three independent experiments (D–F). Data are expressed as the mean ± standard error of the mean of three independent groups (G). N.S., not significant (*P* ≥ 0.05). *P < 0.05, **P < 0.01, and ***P < 0.001. Ra, H37Ra; siCtrl, siControl; UN, untreated control.

ROS regulates autophagy by inhibiting the phosphorylation of the Unc-51-like kinases ULK1 and ULK2 (39 – 41). To investigate the effect of Herp deficiency on autophagy induction, we measured microtubule-associated protein 1A/1B-light chain 3 (LC3) and p62/SQSTM1 levels in WT and Herp-deficient macrophages after mycobacterial infection. Although the production of LC3-II was increased, the production of p62 was decreased in both Herp-depleted BMDMs and Raw264.7 cells compared to the controls (Fig. 6A and B). Protein levels of LC3-II and p62 were similar between uninfected Herp-KO Raw264.7 cells and WT controls (Fig. S6A). Moreover, bafilomycin A1 treatment increased LC3 and p62 levels in a time-dependent manner in H37Ra-infected Herp-KO Raw264.7 cells compared to WT Raw264.7 cells (Fig. 6C). To accurately estimate autophagic activity, LC3 was assessed by flow cytometry or fluorescence microscopy (Fig. 6D, E and F). H37Ra-induced LC3 expression levels were significantly higher in Herp-KO Raw264.7 cells than in the controls (Fig. 6D). In addition, immunofluorescence analyses using an anti-LC3 antibody showed an increase in the colocalization of H37Ra-RFP and LC3 in Herp-KO Raw264.7 cells compared to WT Raw264.7 cells (Fig. 6E). An increased number of LC3-positive autophagic puncta was also detected in H37Ra-infected Herp-KO Raw264.7 cells (Fig. 6F). These observations showed that mycobacteria-induced autophagy induction was stronger in Herp-deficient macrophages than in control macrophages.

To determine whether H37Ra-induced ROS are associated with LC3 induction, LC3 production was evaluated in the presence of the antioxidant N-acetylcysteine (NAC). As expected, the increased LC3 level in Herp-KO macrophages was reduced by NAC treatment (Fig. 6F). To further investigate whether ROS-mediated autophagy in Herp-KO Raw264.7 cells affects the intracellular survival of H37Ra, we examined the impact of autophagy-related 5 (Atg5) in both WT Raw264.7 cells and Herp-KO Raw264.7 cells during H37Ra infection. The decreased survival of H37Ra in Herp-KO Raw264.7 cells was partially reversed by Atg5-knockdown (Fig. 6G). Thus, the relative increase in ROS by Herp depletion may contribute to a robust induction of autophagy during H37Ra infection, which results in the regulation of the intracellular survival of H37Ra.

### Herp depletion suppresses the growth of Mtb *in vivo*

**Fig 7:**
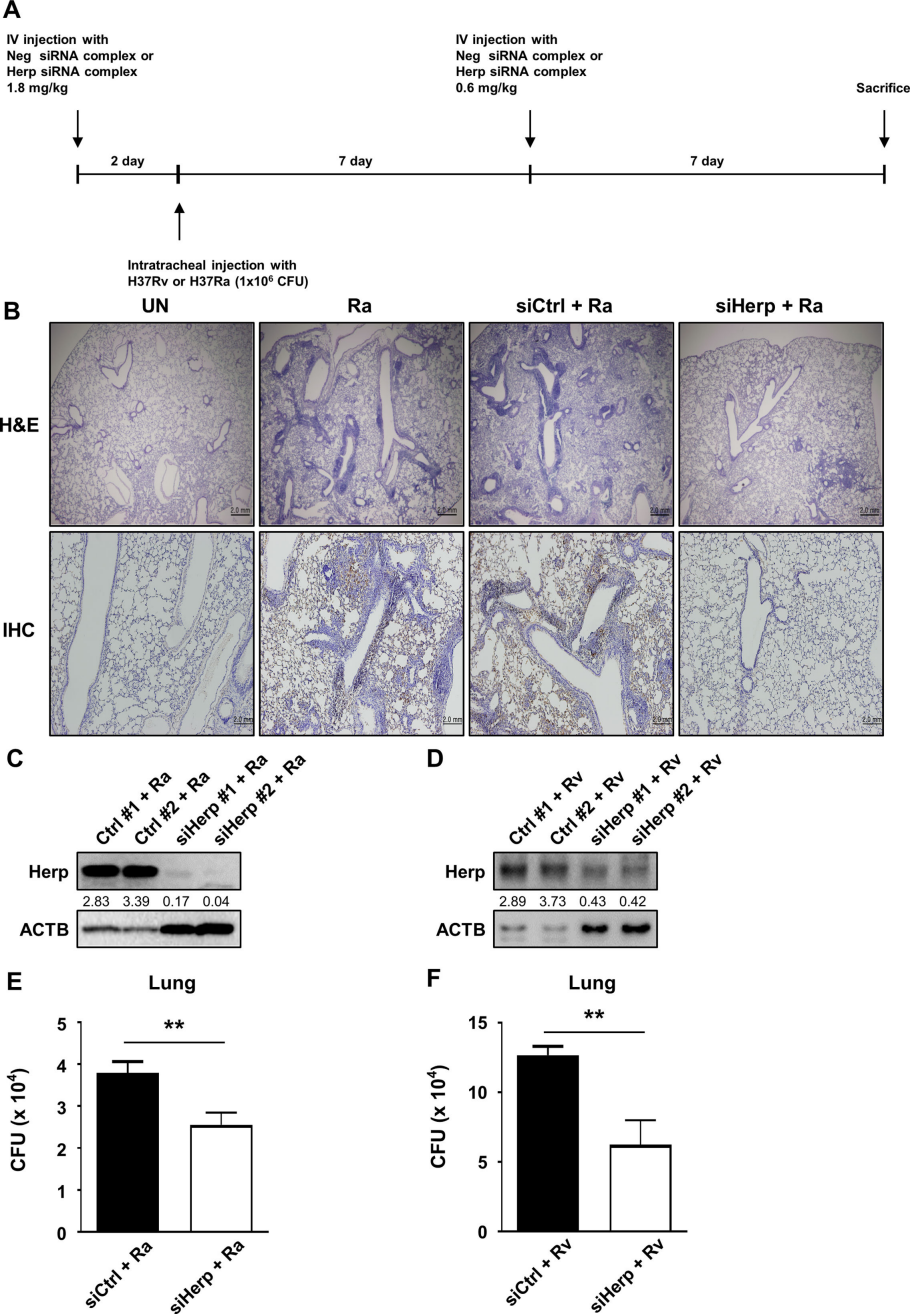
Depletion of Herp suppresses Mtb H37Ra growth *in vivo*. (A) C57BL/6 mice were transfected with siHerp and siControl (1.8 mg/kg) by intravenous injection using Invivofectamine for 2 days and infected with H37Ra or H37Rv (1 × 10^6^ CFU per mouse) via the intratracheal route. Mice were reinjected with siHerp and siControl (0.6 mg/kg) by intravenous injection of Invivofectamine at the indicated times. After 14 days of infection, the mice were sacrificed. (B) Lung inflammation was analyzed using hematoxylin and eosin staining, and HERP expression was analyzed using immunohistochemistry. (C and D) Protein levels in the lungs were analyzed by western blotting. (E and F) The intracellular survival of H37Ra or H37Rv in the lungs was measured using colony-forming unit analysis. The quantification of western blots was performed using ImageJ and normalized by the ACTB density. Data are expressed as the mean ± standard error of the mean of three independent experiments. N.S., not significant (*P* ≥ 0.05). *P < 0.05, **P < 0.01, and ***P < 0.001. IV, intravenous; Ra, H37Ra; UN, untreated control.

To examine the function of Herp in mycobacteria-infected mice, siRNA for Herp was injected into the tail vein every week for 2 weeks at a dose of 1.8 mg/kg/week or a dose of 0.6 mg/kg/2–4 weeks. Herp was then knocked down, and the number of viable bacteria was counted. The mice were infected with H37Ra or H37Rv via intratracheal injection (1 × 10^6^ CFU for 2 weeks, and then sacrificed (Fig. 7A). The levels of Herp in the lungs were knocked down through siHerp injection (Fig. 7C and D). We observed that increased Herp expression by H37Ra infection was successfully reduced in the lungs of siHerp-injected mice (Fig. 7B). Remarkably, we found that H37Ra-induced lung inflammation was significantly reduced in siHerp-injected mice (Fig. 7B). Furthermore, the intracellular survival of mycobacteria (H37Ra or H37Rv) in the lungs of siHerp-injected mice was significantly reduced compared with the control group (Fig. 7E and F). After intratracheal infection, the total number of mycobacteria was similar between H37Rv and H37Ra in the lungs (Fig. S7A). Thus, we conclude that Herp is important for regulating inflammation and bacterial growth during mycobacterial infections.

## DISCUSSION

In this study, we found that H37Ra infection induced Herp production in an ATF6-dependent manner (Fig. 1E). Upon Mtb infection, Herp production rapidly increased at 24 h, subsequently returning to normal levels 48 h after infection (Fig. S1A). These expression patterns are similar to those of other ER stress-sensor molecules, such as BiP and p-eIF2ɑ (Fig. 2C). The UPR is characterized by an increase in ER chaperones and a decrease in the amount of proteins entering the ER, which is caused by the phosphorylation of eIF2ɑ (42). These results show that Mtb infection-induced UPR strongly increased 24 h after infection and gradually decreased until 48 h. Therefore, Herp production is dependent on UPR during Mtb infection. The ER stress response element (ERSE) I and ERSE II sites on the promoter region of Herp can be transactivated by ATF6 binding (43). Unlike the other arms of the UPR, such as PERK and IRE1, the ATF6 arm has not been linked to pro-apoptotic signaling (44). Therefore, ATF6 integrates with multiple stress-responsive signaling pathways to promote the protective and adaptive remodeling of cellular physiological and pathological insults (44). Interestingly, the involvement of ATF6 in the protection of cells is associated with the ability of pathogenic Mtb to protect cells at an early stage of infection (45). Although the role of apoptosis in the pathogenesis and progression of TB remains controversial, there appears to be a consensus that pathogenic Mtb inhibits apoptosis as a survival strategy and facilitates its proliferation within host cells (45). Herp depletion increased the production of ER stress-sensor molecules; however, it did not affect the rate of apoptosis in Mtb-infected macrophages. CHOP production was reduced in the Herp-KO macrophages (Fig. 2C). Previous studies have shown that CHOP-mediated apoptosis plays an important role in the suppression of intracellular mycobacteria (10, 13, 17, 46). Given that ER stress-mediated apoptosis is critical for suppressing the intracellular survival of mycobacteria (10), Herp may play another role in macrophage protection against H37Ra infection. ER stress is known to increase the binding of Herp to HRD1 in ERAD complexes and improve HRD1-dependent ubiquitylation, resulting in the rapid clearance of abnormal proteins (20, 21). IRE1α, a UPR signaling pathway, is a known substrate of SEL1L-HRD1 ERAD, which is a process that depends on HRD1 (47). Herp depletion reduced the production of HRD1 in the H37Ra-infected macrophages (Fig. 2A and B). Given that the PERK and ATF6 signaling pathways are required for the maximal induction of CHOP (48), Herp may play an important role in ER stress-induced CHOP production in H37Ra-infected macrophages. A previous study suggested that crosstalk between UPR and ERAD is critical under physiological and pathological conditions for ER homeostasis (33). Although it is difficult to elucidate whether Herp is induced by H37Ra or macrophages, Herp-mediated ER quality control plays an important role in regulating the intracellular survival of H37Ra.

Our data showed that ROS and the production levels of gp91-phox in Herp-depleted macrophages were higher than in the control during H37Ra infection (Fig. 4B and C). Although ER stress contributes to ROS generation (49), 4-phenylbutyrate pretreatment did not significantly decrease ROS levels in Herp-depleted macrophages (Fig. 4A). It is likely that the production levels of ER stress sensor molecules in Herp-depleted macrophages are insufficient to induce ROS synthesis. ROS production is dependent on the activity of the phagocyte NOX2 during perturbation of ubiquitin homeostasis (50) and the ERAD pathway regulates the protein levels of NOX2 in BMDMs (51). Therefore, we suggest that ERAD perturbation upregulates NOX2 production, leading to ROS production in Herp-depleted macrophages during H37Ra infection. It is also important to consider that the influx of Ca^2+^ from the ER to mitochondria is increased by ITPR, which is regulated by Herp (52). Furthermore, a previous report suggested that overload of mitochondrial matrix Ca^2+^ leads to increased ROS production (37). We showed that the inhibition of ITPR by Xestospongin C decreased mROS only in Herp-KO Raw264.7 cells (Fig. 5A). As indicated by the stronger increase in cytosolic Ca^2+^ levels in Herp-KO Raw264.7 cells compared to WT controls during H37Ra infection (Fig. S5B and S5C), it is possible that ITPR-mediated mROS production is significantly impacted in Herp-KO macrophages. Herp interacted with NOX2 and ITPR (Fig. 5B). Given that the binding of Herp to HRD1 regulates the activity of HRD1-containing ERAD complexes (21), Herp depletion in H37Ra-infected macrophages may contribute to ROS generation through increased levels of NOX2 components and ITPR via the ERAD pathway.

Here, we showed that the downregulation of Herp in H37Ra-infected macrophages leads to suppression of the intracellular growth of H37Ra (Fig. 3A and B). It is likely that ER stress-mediated apoptosis in Herp-KO macrophages is not involved in mycobacterial growth inhibition (Fig. 3C). However, the regulation of the intracellular survival of Mtb by Herp remains elusive. We showed that the production of LC3-II increased and that the production of p62 decreased in Herp-depleted macrophages during H37Ra infection (Fig. 6A and B). It is well known that autophagy plays a crucial role in eliminating mycobacteria in macrophages (53). A previous study demonstrated that autophagy-related protein 5 and BECLIN-1 were increased in Herp-depleted cells compared to WT controls, which sequentially leads to activation of protective autophagy for cell survival during glucose starvation (54). Therefore, the increased level of LC3 in Herp-depleted macrophages may suppress the intracellular survival of H37Ra through the autophagy pathway. According to a previous study, excessive accumulation of ROS can disrupt cellular homeostasis, leading to oxidative stress, mitochondrial dysfunction, and the induction of autophagy (55). Notably, our data showed that NAC treatment resulted in a comparable number of LC3 puncta in Herp-KO Raw264.7 cells and WT controls during H37Ra infection (Fig. 6F). Thus, these data suggest that the suppression of Herp expression increases autophagy through ROS production mediated by both gp91-phox and ITPR, while decreasing intracellular survival of H37Ra through increased autophagy.

Both H37Rv and H37Ra infection induced Herp production in macrophages (Fig. S1A), and mycobacterial survival in Herp-knockdown was inhibited *in vivo* (Fig. 7E and F). The level of Herp production induced by H37Ra was higher than that of H37Rv (Fig. S1A). This difference may be related to the different ER stress responses between H37Rv and H37Ra infection, as H37Ra-infected macrophages strongly induced ER stress-associated molecules compared to H37Rv (8). Although we did not compare the role of Herp in macrophages infected with H37Rv and H37Ra, our results suggest that Herp plays an important role in mycobacteria-infected macrophages.

In summary, Herp was induced by H37Ra-mediated ATF6 production in macrophages. Herp depletion upregulated ROS levels via gp91-phox and ITPR production in H37Ra-infected macrophages. Increased levels of ROS inhibited H37Ra growth by inducing autophagy in Herp-depleted macrophages. The suppressive effect of siHerp on the intracellular survival of mycobacteria in macrophages suggests that Herp is one of the therapeutic targets for tuberculosis.

## MATERIALS AND METHODS

### Cell culture

Murine macrophage RAW 264.7 cells were cultured in Dulbecco’s modified Eagle’s medium supplemented with 10% heat-inactivated fetal bovine serum and penicillin-streptomycin (100 mg/mL). Cell cultures were maintained at 5% CO_2_ and 37°C. Primary BMDMs were isolated from C57BL/6 mice (6–8 weeks old) and differentiated by supplementation with macrophage colony-stimulating factor (25 ng/mL; R&D Systems) for 4 days.

### Ethics

WT mice (C57BL/6 background) aged 6–10 weeks were used in the experiments (Nara-Biotech). All animal-related procedures were reviewed and approved by the Institutional Animal Care and Use Committee, Chungnam National University, College of Medicine (Daejeon, Korea, CNU-00907). All animal experiments were performed in accordance with the Korean Food and Drug Administration guidelines.

### Bacterial culture, infection, and intracellular survival analysis *in vitro* and *in vivo*

The Mtb strain H37Ra (ATCC 25177) was obtained from the American Type Culture Collection. H37Ra were cultured in Middlebrook 7H9 liquid medium supplemented with 10% OADC (oleic acid, albumin, dextrose, and catalase) and 5% glycerol. Bacterial cultures were spun at low speed (140 rpm) at 37°C until they reached the log phase (OD_600_ 0.6–0.8). The bacteria were harvested by centrifugation at 3,000 × *g* for 30 min, washed, and resuspended in phosphate-buffered saline (PBS). They were then briefly sonicated (a few seconds) before infection. The cells were infected with H37Ra at an MOI of 5:1 for 3 h. To remove non-phagocytized bacteria, the cells were washed and cultured in medium. The cells were then lysed in autoclaved distilled water to allow the collection of intracellular bacteria. Lysates were plated separately on Middle Brook 7H10 agar plates and incubated at 37°C for 14 or 21 days. Heat-killed H37Ra was prepared by heating H37Ra in PBS at 80°C for 30 min. For the *in vivo* bacterial infection study, we used 8-week-old female mice. The mice were infected by intratracheal infection with 1 × 10^6^ CFU of H37Rv (ATCC 25618) or H37Ra. The mice were sacrificed 2 weeks later. The right lung of each mouse was obtained for intracellular survival analysis. To measure protein levels in the lungs, the left lung of each mouse was isolated and homogenized in RIPA buffer.

### Reagents

Chemicals and inhibitors, such as Ceapin-A7 (Millipore), Irestatin (Axon), GSK2606414 (Millipore), 4-phenylbutyrate (Millipore), and tunicamycin (Millipore), were dissolved in dimethyl sulfoxide and diluted to the desired concentration using the culture medium.

### Western blot analysis

Western blotting was performed as described previously (56). The primary antibodies used were as follows: anti-Herpud1, anti-CHOP, anti-phospho-eIF2a, anti-eIF2a, anti-GRP78/BiP, anti-LC3, anti-ACTB (Cell Signaling), anti-HRD1, anti-ITPR (Invitrogen), anti-gp91phox, and p62 (Santa Cruz Biotechnology). The secondary antibodies used were anti-rabbit IgG-HRP (Cell Signaling) and anti-mouse-IgG-HRP (Calbiochem). The quantification of western blots was performed using ImageJ software (v1.53) and normalized by the ACTB density.

### Apoptosis analysis

The cells were analyzed by flow cytometry using a FACS Canto II cytometer (BD Biosciences). The cells were stained using an Annexin-V/PI staining kit according to the manufacturer’s instructions. Flow cytometry data were collected and analyzed using FlowJo software (Tree Star).

### Measurement of ROS and mitochondrial superoxide

Intracellular ROS levels were measured using the dihydroethidium assay, and the mROS levels were measured with MitoSOX (Invitrogen). The cells were washed with PBS and incubated with dihydroethidium (20 µM) or MitoSOX (2 µM) for 30 min at 37°C. They were then washed three times with PBS and fixed with 4% paraformaldehyde. The samples were analyzed using a FACS Canto II cytometer. The data were processed using FlowJo software.

### Measurement of intracellular Ca^2+^

The intracellular Ca^2+^ levels were assessed with a Fluo-4-AM (Invitrogen). The cells were washed with PBS and incubated with Fluo-4-AM (5 µM) for 30 min at 37°C. After three washes with PBS, the cells were fixed with 4% paraformaldehyde. Samples were analyzed using a FACS Canto II cytometer or a ZEISS DP70 fluorescence microscope (ZEISS). The data were processed using FlowJo software or ZEN software. The fluorescent intensity in a fixed region of interest adjacent to H37Ra-GFP positive cells was measured using ZEN software. Each condition was analyzed in triplicate, and at least 30 cells per well were counted.

### Reverse transcription-polymerase chain reaction

Total RNA was extracted from cells using TRIzol (Invitrogen) according to the manufacturer’s instructions. After RNA quantitation, cDNA was synthesized using a reverse transcriptase premix (Elpis Biotech). PCR was performed using Prime Taq Premix (GeNet Bio). The samples were viewed using Gel-Doc (Davinch-K).

### Autophagy analysis

To quantify autophagy, LC3 punctate dots were identified using ImageJ, V1.53. The colocalization coefficients were calculated by summing the pixels in the colocalized region (GFP + RFP) and dividing the sum of the pixels in the RFP. Each condition was assayed in triplicate, and at least 100 cells per well were counted.

### Transfection of small interfering RNA

Gene silencing was performed by the small interfering RNA (siRNA) technique using siRNAs (200 nM) targeting mRNA (Bioneer) and negative control siRNAs (Bioneer). The siRNAs were transfected into cultured cells using Lipofectamine 3000 (Invitrogen) according to the manufacturer’s instructions. At 5-h post-transfection, the cells were cultured in fresh complete medium for 24 h.

### PLA procedure

The PLA assay was performed according to the manufacturer’s instructions using the following primary antibodies: anti-HRD1 (Invitrogen), anti-gp91-phox (Santa Cruz Biotechnology), anti-ITPR (Invitrogen), anti-Herp (Novusbio), and anti-Herp (Invitrogen). Images were taken using a ZEISS DP70 fluorescence microscope. The data were processed using ZEN software. PLA punctate in H37Ra-GFP positive cells was counted using ZEN software. Each condition was analyzed in triplicate, and at least 30 cells per well were counted.

### Immunofluorescence

Infected cells were fixed in 4% paraformaldehyde and incubated overnight with a primary antibody targeting LC3, followed by incubation with a secondary antibody (Alexa Fluor 488 anti-rabbit IgG; Life Technologies) for 2 h at room temperature. Cell nuclei were visualized using DAPI staining. Antibody- and DAPI-stained cells were detected using a ZEISS DP70 fluorescence microscope with excitation lasers and cameras associated with a set of filters covering a detection wavelength ranging from 400 to 700 nM. DAPI-stained nuclei were detected using a 405 nm. Green or red signals corresponding to LC3, Fluo-4-AM, PLA puncta, or H37Ra-RFP were recorded using 488 or 561 nm lasers, respectively. The data were processed using ZEN software.

### Establishment of transgenic Raw264.7 cell lines

To generate the Herp-KO Raw264.7 cell line, we selected guide RNA targeting the Herp region, based on the web design tool (http://chopchop.cbu.uib.no/#). One validated guide RNA, along with Cas9 mRNA, was introduced into Raw264.7 cells using Lipofectamine 3000. The resulting genomic DNA was screened for deletions in the target region by PCR and Sanger sequencing.

### Hematoxylin and eosin staining and immunohistochemistry

After formaldehyde fixation, dehydration, and paraffin embedding, the tissues were cut into 5 mM sections on a microtome. The lung slide was stained with hematoxylin and eosin and anti-Herp antibody. Images were taken using a fluorescence microscope (Olympus).

### Statistical analyses

Each experiment was performed at least three times, and representative results were presented. Statistical significance was analyzed using GraphPad Prism 5 and the Mann–Whitney *U* test. Statistical significance is indicated by *P < 0.05, **P < 0.01, and ***P < 0.001.

## SUPPLEMENTAL MATERIAL

Figure S1 mbio.01535-23-s0001.tif Herp is induced during mycobacterial infection.

Figure S2 mbio.01535-23-s0002.tif HRD1, CHOP, BiP, p630 eIF2ɑ, and eIF2ɑ levels.

Figure S3 mbio.01535-23-s0003.tif Levels of ROS and apoptosis.

Figure S4 mbio.01535-23-s0004.tif gp91-phox and ITPR levels.

Figure S5 mbio.01535-23-s0005.tif Herp plays a key role in the release of Ca^2+^ in Herp-KO Raw264.7 cells.

Figure S6 mbio.01535-23-s0006.tif LC3 and p62 levels.

Figure S7 mbio.01535-23-s0007.tif Mtb H37Ra and Mtb H37Rv growth *in vivo*.

ASM does not own the copyrights to Supplemental Material that may be linked to, or accessed through, an article. The authors have granted ASM a non-exclusive, world-wide license to publish the Supplemental Material files. Please contact the corresponding author directly for reuse.
