# Supplementary figures and images for: Herp regulates intracellular survival of Mycobacterium tuberculosis H37Ra in macrophages by regulating reactive oxygen species-mediated autophagy
Source: mBio. 2023 Oct 6;14(5):e01535-23. doi: 10.1128/mbio.01535-23 (PMC10653826; doi:10.1128/mbio.01535-23)

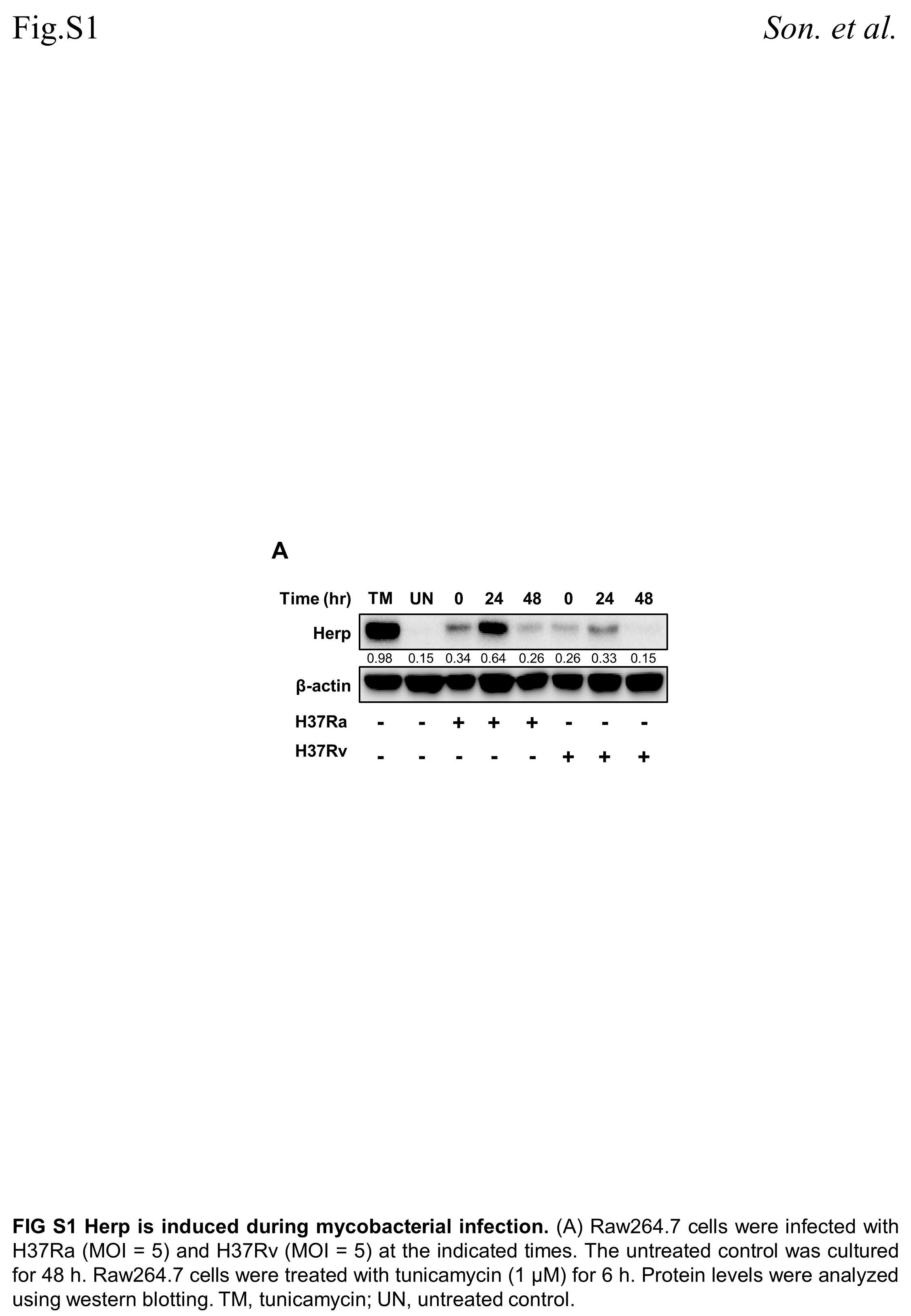

Supplement: Figure S1 — Herp is induced during mycobacterial infection. [file mbio.01535-23-s0001.tif]

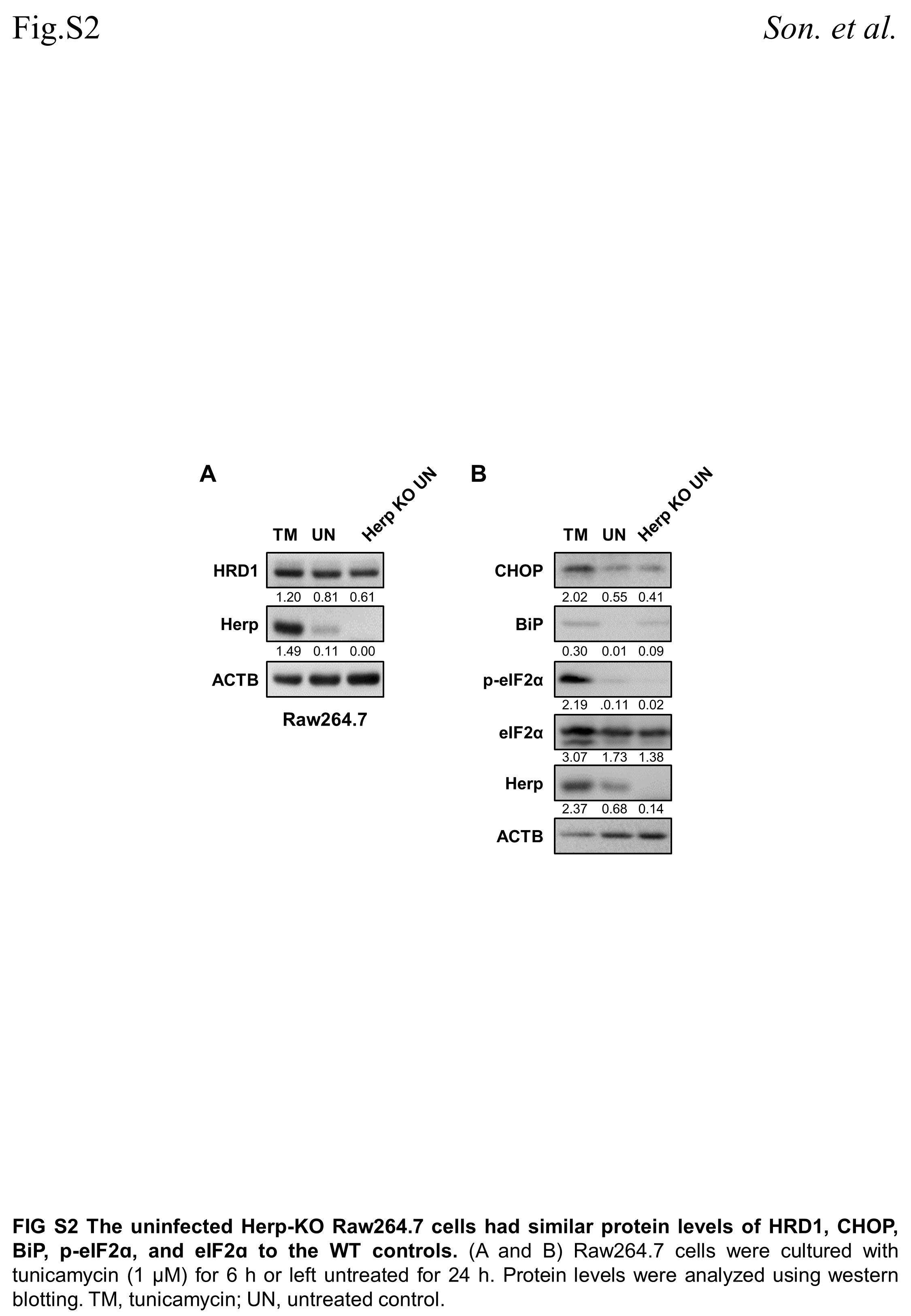

Supplement: Figure S2 — HRD1, CHOP, BiP, p630 eIF2ɑ, and eIF2ɑ levels. [file mbio.01535-23-s0002.tif]

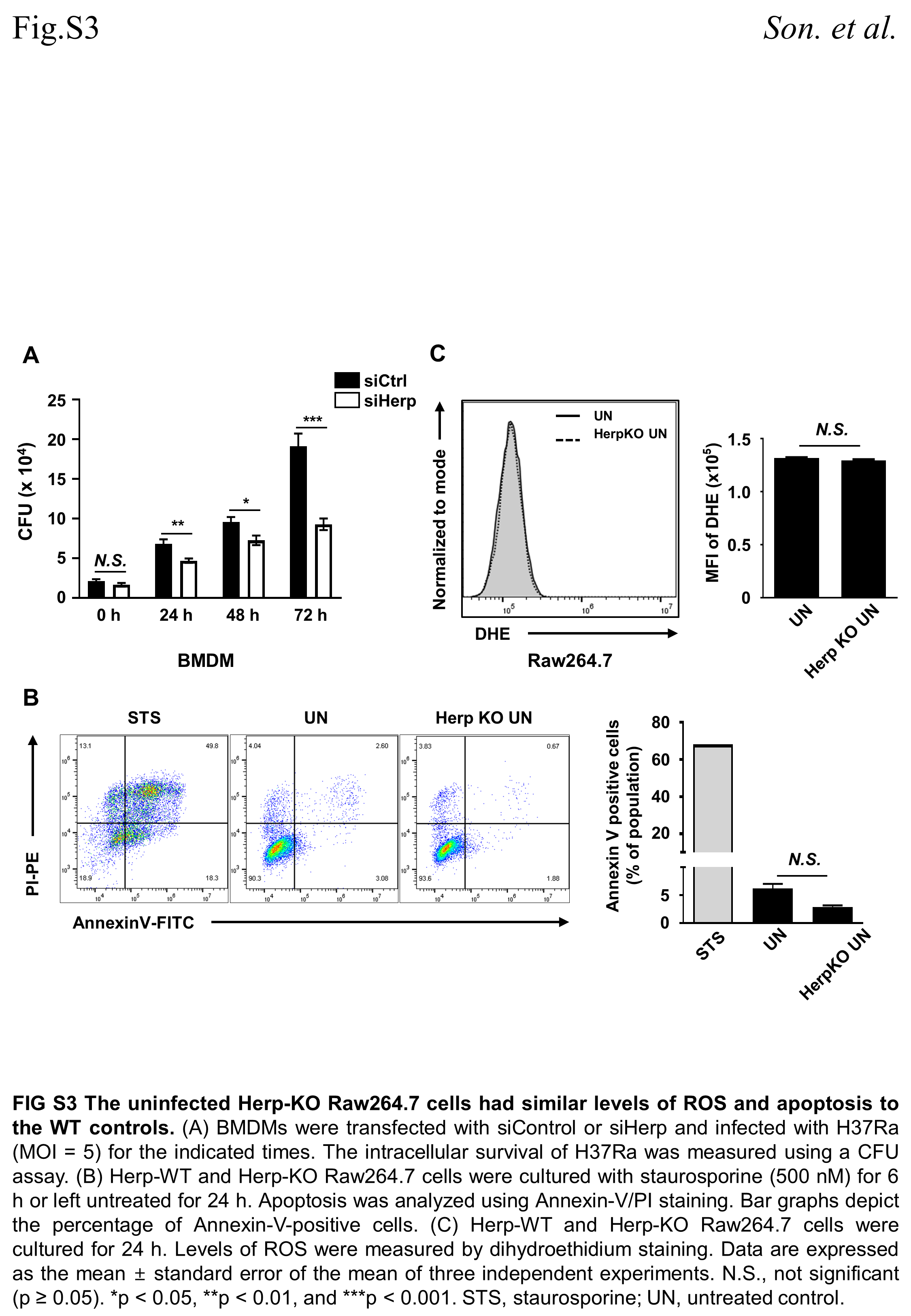

Supplement: Figure S3 — Levels of ROS and apoptosis. [file mbio.01535-23-s0003.tif]

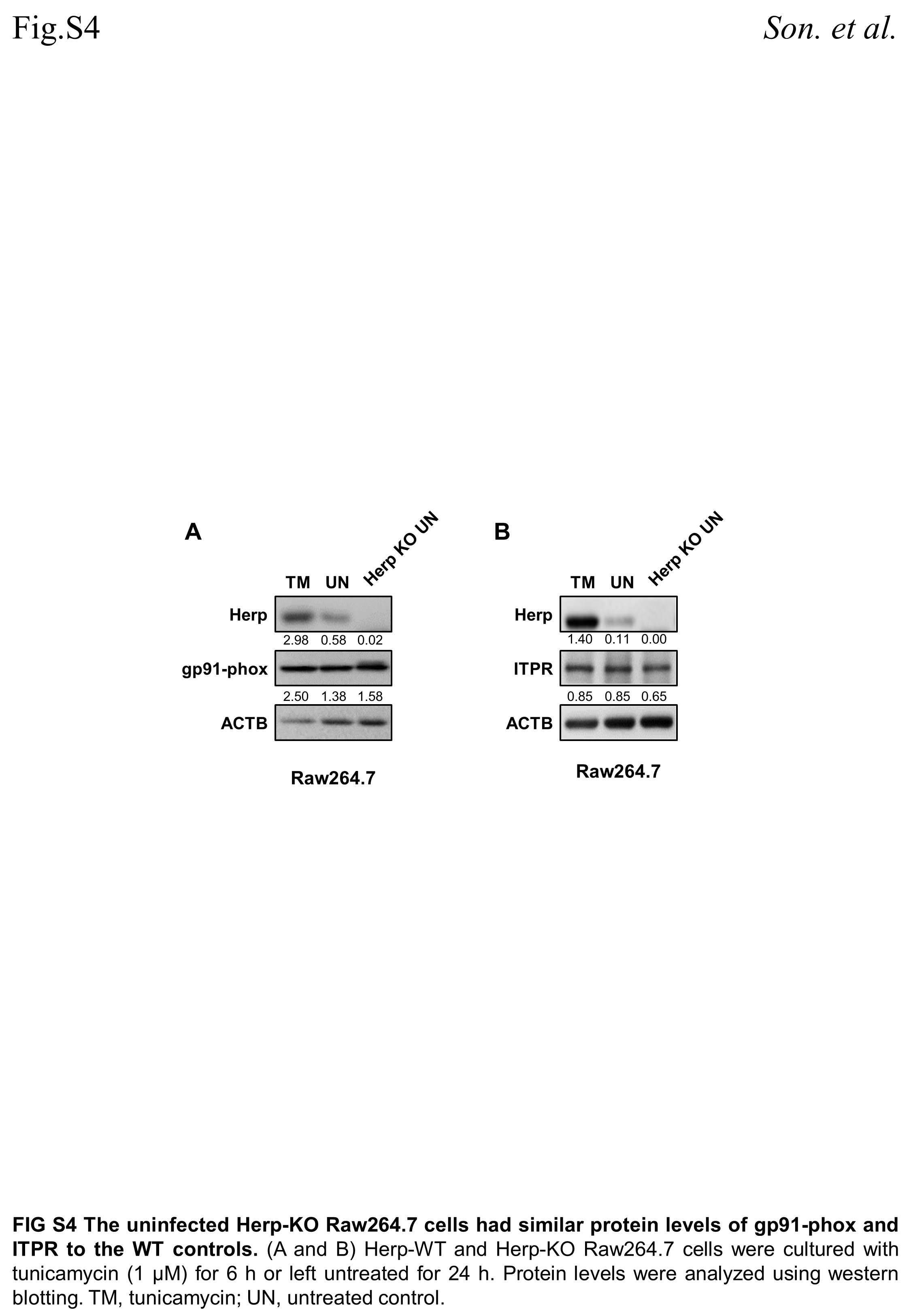

Supplement: Figure S4 — gp91-phox and ITPR levels. [file mbio.01535-23-s0004.tif]

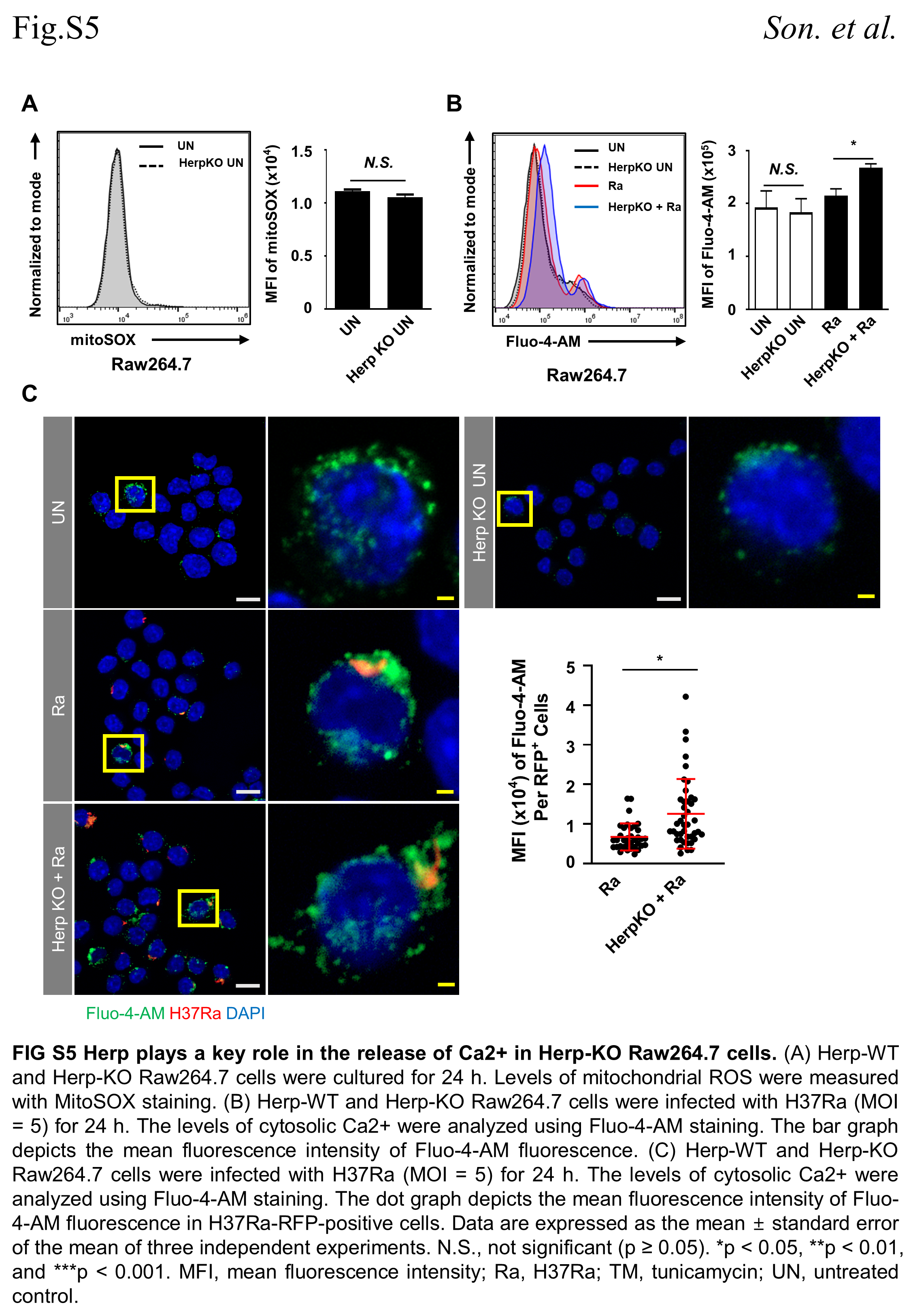

Supplement: Figure S5 — Herp plays a key role in the release of Ca2+ in Herp-KO Raw264.7 cells. [file mbio.01535-23-s0005.tif]

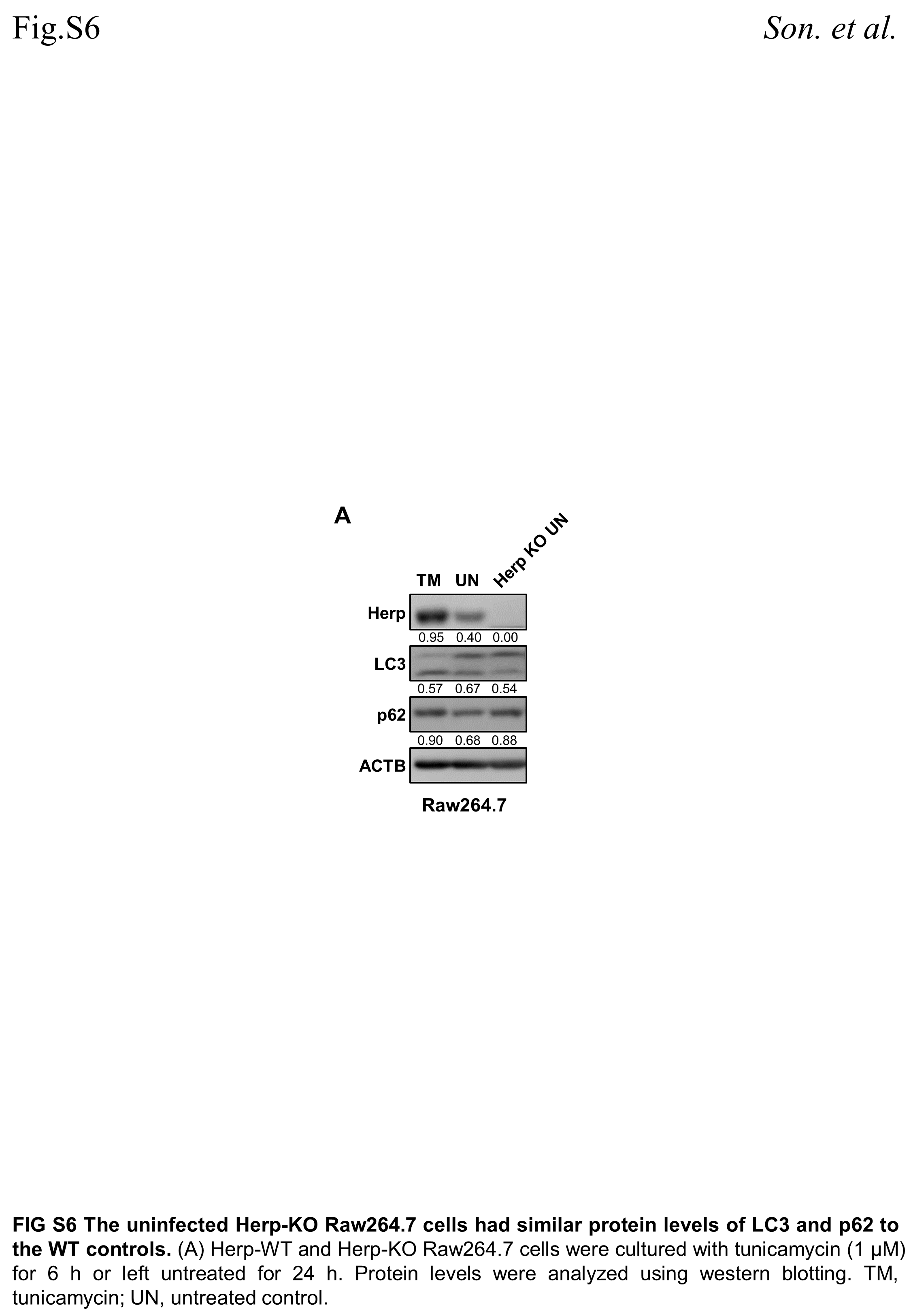

Supplement: Figure S6 — LC3 and p62 levels. [file mbio.01535-23-s0006.tif]

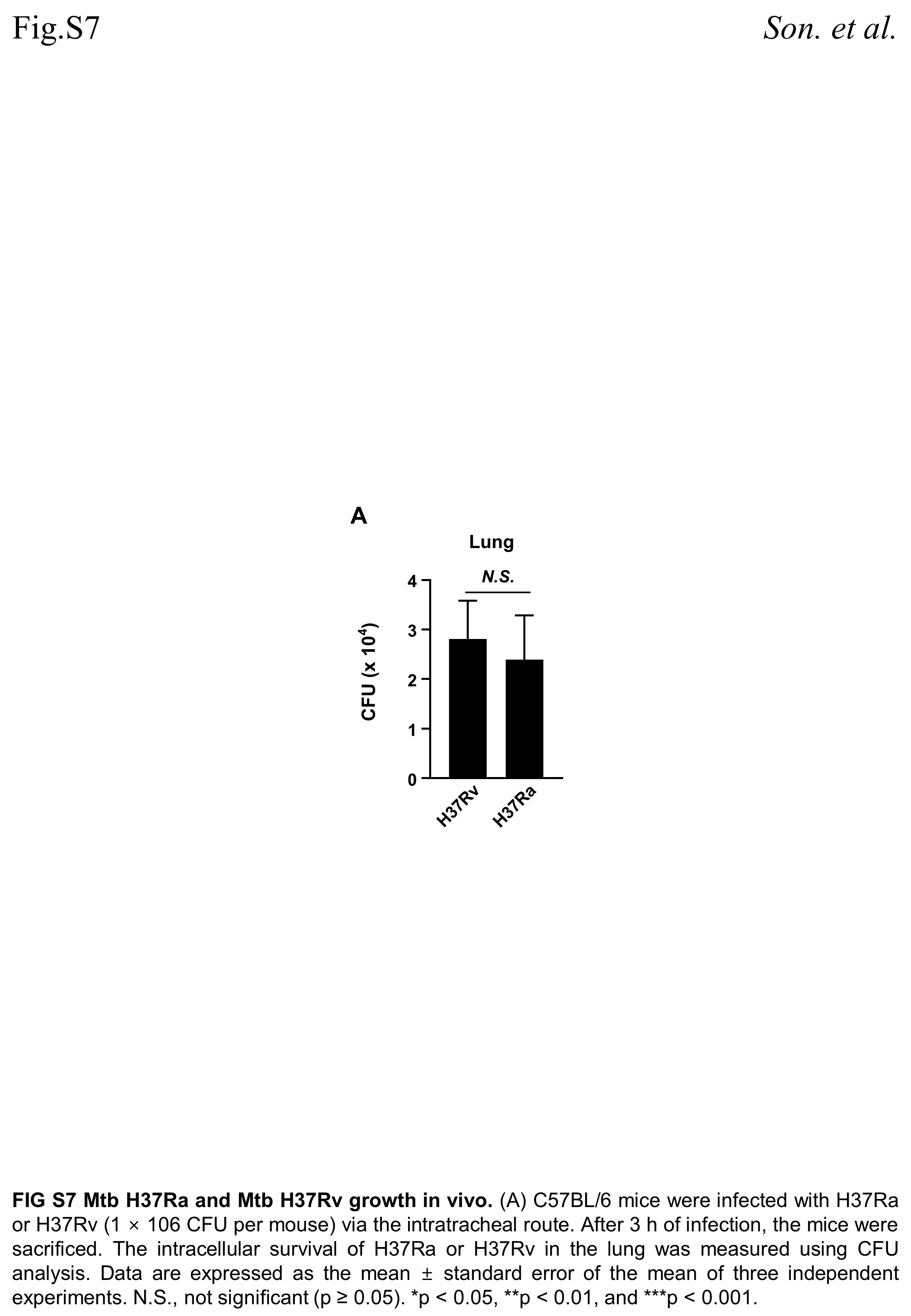

Supplement: Figure S7 — Mtb H37Ra and Mtb H37Rv growth in vivo. [file mbio.01535-23-s0007.tif]
